# Time Course, Distribution and Cell Types of Induction of Transforming Growth Factor Betas following Middle Cerebral Artery Occlusion in the Rat Brain

**DOI:** 10.1371/journal.pone.0046731

**Published:** 2012-10-08

**Authors:** Gabriella Pál, Csilla Vincze, Éva Renner, Edina A. Wappler, Zoltán Nagy, Gábor Lovas, Arpád Dobolyi

**Affiliations:** 1 Neuromorphological and Neuroendocrine Research Laboratory, Department of Anatomy, Histology and Embryology, Semmelweis University and the Hungarian Academy of Sciences, Budapest, Hungary; 2 Department of Neurology, Semmelweis University, Budapest, Hungary; 3 Cardiovascular Center, Department Section of Vascular Neurology, Semmelweis University, Budapest, Hungary; 4 Department of Anesthesiology and Intensive Therapy, Semmelweis University, Budapest, Hungary; 5 Department of Neurology, Jahn Ferenc Teaching Hospital, Budapest, Hungary; University of Queensland, Australia

## Abstract

Transforming growth factor-βs (TGF-β1–3) are cytokines that regulate the proliferation, differentiation, and survival of various cell types. The present study describes the induction of TGF-β1–3 in the rat after focal ischemia at 3 h, 24 h, 72 h and 1 month after transient (1 h) or permanent (24 h) middle cerebral artery occlusion (MCAO) using *in situ* hybridization histochemistry and quantitative analysis. Double labeling with different markers was used to identify the localization of TGF-β mRNA relative to the penumbra and glial scar, and the types of cells expressing TGF-βs. TGF-β1 expression increased 3 h after MCAO in the penumbra and was further elevated 24 h after MCAO. TGF-β1 was present mostly in microglial cells but also in some astrocytes. By 72 h and 1 month after the occlusion, TGF-β1 mRNA-expressing cells also appeared in microglia within the ischemic core and in the glial scar. In contrast, TGF-β2 mRNA level was increased in neurons but not in astrocytes or microglial cells in layers II, III, and V of the ipsilateral cerebral cortex 24 h after MCAO. TGF-β3 was not induced in cells around the penumbra. Its expression increased in only a few cells in layer II of the cerebral cortex 24 h after MCAO. The levels of TGF-β2 and -β3 decreased at subsequent time points. Permanent MCAO further elevated the levels of all 3 subtypes of TGF-βs suggesting that reperfusion is not a major factor in their induction. TGF-β1 did not co-localize with either Fos or ATF-3, while the co-localization of TGF-β2 with Fos but not with ATF-3 suggests that cortical spreading depolarization, but not damage to neural processes, might be the mechanism of induction for TGF-β2. The results imply that endogenous TGF-βs are induced by different mechanisms following an ischemic attack in the brain suggesting that they are involved in distinct spatially and temporally regulated inflammatory and neuroprotective processes.

## Introduction

Three separate genes encode TGF-β1, -β2, and -β3 [1], [2]. TGF-βs affect cell proliferation, differentiation, and extracellular matrix formation in a variety of tissues [3] by means of serine-threonine kinase domain-containing TGF-β receptors [4], [5]. In the normal brain, TGF-β1 immunoreactivity is present in the epithelial and meningeal cells of the choroid plexus [6]. *In situ* hybridization histochemistry also indicated the expression of TGF-β1 in some restricted brain regions [7]. TGF-β2 and -β3 were constitutively present in several brain regions as, confirmed at the mRNA [7] and protein levels [6], and their expression patterns greatly overlapped, although the expression level of TGF-β2 was considerably higher than that of TGF-β3 [8]. In the cerebral cortex, TGF-β2 expression was very intense in layer V Layers III and IV also contained TGF-β2 and –β3, respectively, while TGF-βs were absent in the caudate putamen [6], [7].

Among the potential neural functions of the TGF-βs, their involvement in neuroprotection is the most established [8]–[15]. The administration of TGF-β1 into the brain reduced infarct size in experimental models of ischemia [16]–[18], while antagonizing the endogenous actions of TGF-β1 with an injection of a soluble TGF-β type II receptor, which binds TGF-β1 and prevents its biological actions, resulted in a dramatic increase in infarct area [19]. Although the involvement of other subtypes of TGF-βs in ischemia has not been established, their neuroprotective actions are conceivable based on their involvement in the survival of midbrain dopaminergic neurons [20]–[22] and motoneurons [23] and in the modulation of plasticity in hippocampal neurons [24]. In addition to neuroprotection, TGF-βs may also participate in regenerative processes, as they are known to increase neurogenesis in the adult dentate gyrus [25], and subventricular zone [26] and promote a neuronal cell fate for cortical and hippocampal progenitors [27].

The induction of TGF-β1 has been investigated following middle cerebral artery occlusion (MCAO), a model of focal ischemia [28]. An increase in TGF-β1 mRNA in the cingulate cortex as an early event was followed by the induction of TGF-β1 within the infarct area [29], [30]. Delayed expression of TGF-β1 has also been shown by Northern hybridization [31]. Activated microglia and invading macrophages appearing within the infarct area [32] were suggested to be the major sources of TGF-β1 after MCAO [33]. The induction of other subtypes of TGF-βs is also conceivable given that different TGF-βs were induced in necrotizing brain lesions [34] and that the expression of TGF-β2, but not TGF-β1, was correlated with the deposition of scar tissue in lesioned spinal cord [35]. In fact, our previous qualitative report on the induction of TGF-βs 24 h following transient MCAO suggested their involvement in brain ischemia [7]. In the present study, we addressed the following questions to describe the time course, distribution, and cell types involved in the induction of TGF-βs following MCAO and to understand the mechanisms of their induction: 1. How are TGF-β1, -β2 and -β3 mRNAs distributed following 1 h transient and permanent (24 h) MCAO in the rat brain? The penumbra was visualized using immunolabeling of heat shock protein 70 (Hsp70) as previously described [36], [37]. 2. What is the time course of induction of TGF-β1, -β2 and -β3 mRNA following MCAO? *In situ* hybridization histochemistry, which we previously used to describe the distribution of all 3 TGF-βs in the normal brain [7], was used at 3 h, 24 h, 72 h, and 1 month after MCAO to address this question and was followed by a quantitative analysis of the number of TGF-β-expressing cells and the mRNA levels in individual cells. 3. Which cell types express the different subtypes of TGF-βs following MCAO and what is the mechanism of their induction? *In situ* hybridization histochemistry in combination with immunolabeling for neuronal (NeuN), astrocyte (glial fibrillary acidic protein – GFAP), and microglial (ionized calcium-binding adapter molecule 1 - Iba1) markers was applied to identify cells that expressed the 3 different subtypes of TGF-βs in the ischemic rat brain. 4. What mechanisms activate TGF-βs following ischemia? TGF-βs were double stained with the immediate early genes Fos and activating transcription factor-3 (ATF-3), markers of neuronal activation and axonal damage, respectively [38], to establish the involvement of these processes in the induction of TGF-βs.

## Materials and Methods

### Ethics Statement

The Animal Examination Ethical Council of the Animal Protection Advisory Board at Semmelweis University, Budapest specifically approved this study. Thus, the procedures involving rats were carried out according to experimental protocols that meet the guidelines of the Animal Hygiene and Food Control Department, Ministry of Agriculture, Hungary, which is in accordance with EU Directive 2010/63/EU for animal experiments. A total of 48 adult, male Wistar rats (300–450 g body weight; Charles Rivers Laboratories, Hungary) were used in this study. All efforts were made to minimize the number of animals used, and their suffering. The animals were kept in standard laboratory conditions with 12-h light, 12-h dark periods (lights on at 6.00 a.m.), and were supplied with dry rat food and drinking water *ad libitum*. The rats were maintained 3 per cage at a temperature of 22±1°C and anaesthetized with an intramuscular injection (0,2 ml/300 g body weight) of ketamine (60 mg/ml) and xylazine (8 mg/ml) for middle cerebral artery occlusion, transcardial perfusion, and decapitation.

**Figure 1:**
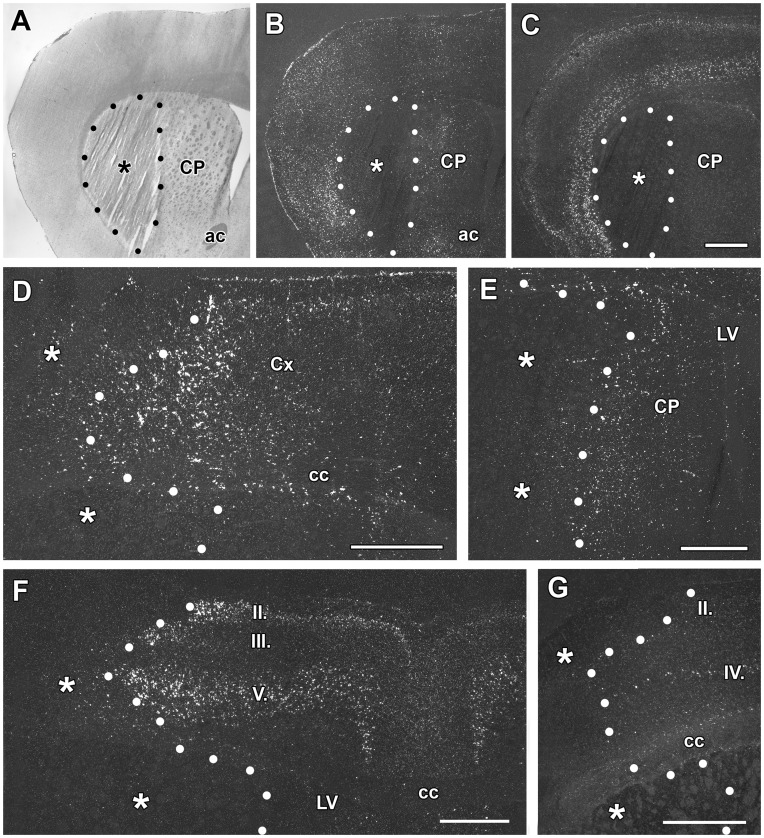
The induction of TGF-β1, -β2, and –β3 mRNA at 3 h and 24 h after middle cerebral artery occlusion (MCAO). Dark-field photomicrographs of *in situ* hybridization sections show the expression of TGF-β mRNAs. The lesion sites are indicated by the star symbols (*) and the borders of the lesions are demarcated by black and white dots. A: Giemsa counterstaining demonstrates the position of the lesion in the caudate putamen 3 h after MCAO. B: The same field of the same section is shown in a dark field image. TGF-β1 mRNA is induced around the lesion. C: TGF-β2 mRNA is present ipsilateral to the lesion. The normal distribution of TGF-β2 can be observed in layers II and V of the cerebral cortex without any apparent increase in the expression level. D: TGF-β1 expression is induced around the lesion in the cerebral cortex 24 h following MCAO. E: TGF-β1 expression is also induced in the peri-infarct area of the caudate putamen. F: TGF-β2 is induced in layers II, III, and V of the ipsilateral cerebral cortex. G: TGF-β3 has a somewhat elevated expression level in some cells of layer II of the cerebral cortex. TGF-β3 is normally present in layer IV of the cerebral cortex and is not induced in this location. Abbreviations: ac – anterior commissure, cc – corpus callosum, CP - caudate putamen, LV - lateral ventricle. Scale bars = 1 mm.

### Middle Cerebral Artery Occlusion

Focal ischemia was induced using a modified intraluminal suture method described previously [39]. Briefly, the left common, internal and external carotid arteries were exposed through a midline incision in the neck and then carefully dissected from the surrounding tissues under an operating microscope. After electrocoagulation of the external and common carotid arteries, a 3-0 silicon rubber-coated monofilament (Doccol, Redlands, CA) was inserted through the common carotid artery into the internal carotid artery to 18 to 20 mm beyond the carotid bifurcation at the base of the middle cerebral artery. The pterygopalatine branch of the internal carotid artery was exposed before the insertion to prevent the filament from entering into the pterygopalatine. An atraumatic aneurysm clip (Codman, Johnson and Johnson, Le Locle, NE, Switzerland) was placed on the internal carotid artery to prevent bleeding. The clip and the monofilament were removed 1 h later for transient ischemia and left in place for 24 h for permanent ischemia. The incision was then sutured. After the required survival period, the brains of the rats intended for single *in situ* hybridization histochemistry were dissected and cut coronally at 1 mm rostral to bregma. The anterior parts of the brains were stained with 2,3,5-triphenyltetrazolium chloride (TTC) and the posterior parts were frozen for *in situ* hybridization histochemistry. The rats intended for double labeling with *in situ* hybridization histochemistry and immunohistochemistry were transcardially perfused before their brains were removed.

**Figure 2:**
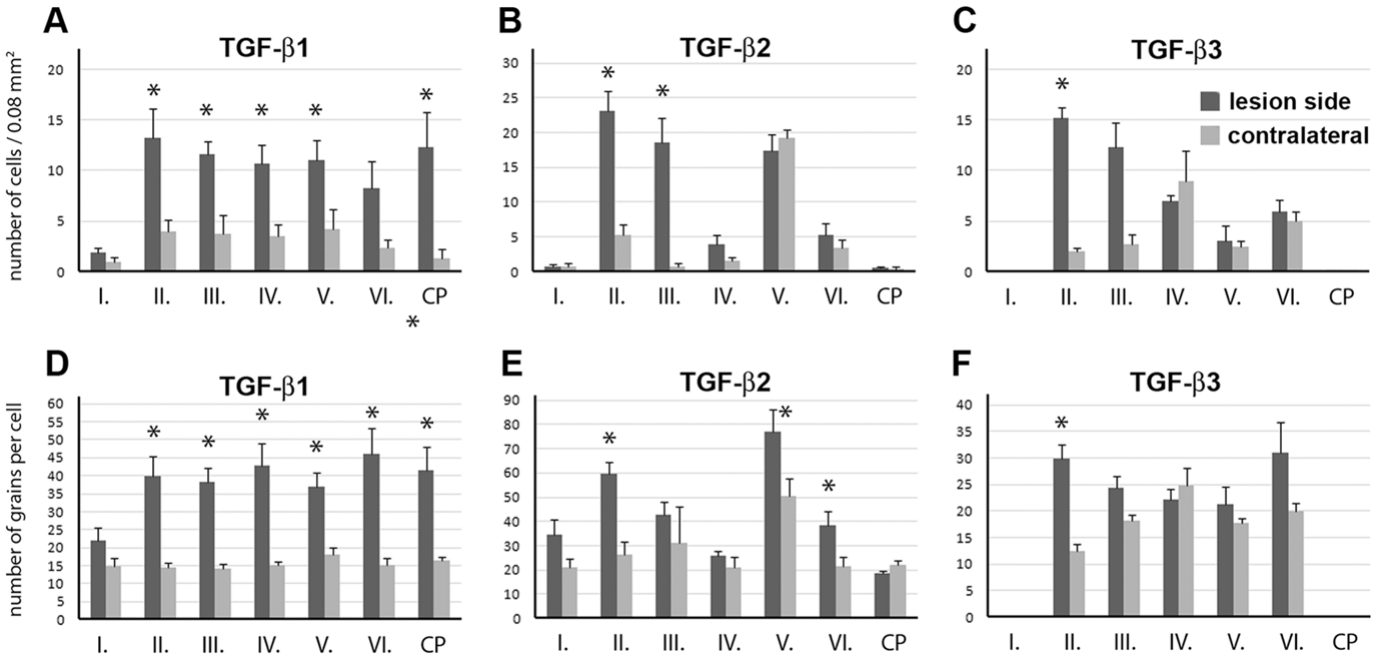
The expression of TGF-β proteins in different brain regions around an ischemic lesion at 24 h following a 1 h transient MCAO. The number of cells was counted in 200×400 µm areas (0.08 mm^2^) of coronal brain sections in cortical layers I-VI and in the caudate putamen (CP). The number of autoradiography grains was counted above TGF-β-positive cell nuclei indicated by the accumulation of autoradiography grains. Measurements were performed around the ischemic lesion (dark gray) and in the corresponding brain area on the contralateral side of the brain (light gray). Sections from 5 animals were involved in the analysis. A–C: The number of cells expressing TGF-β1, -β2, and -β3 in 0.08 mm^2^. D-F: The number of autoradiography grains proportional to the mRNA level of TGF- β1, -β2, and -β3 in single cells. The values around the ischemic lesion and in the corresponding brain area on the contralateral side of the brain were compared using paired Students t-tests, where data obtained from the same section formed the pairs to eliminate variation from the labeling intensity of an individual staining. The star symbol (*) indicates brain regions, in which the number of TGF-β-expressing cells or the mRNA level of the particular subtype of TGF-β in single cells was significantly (p<0.05) elevated.

### Allocation of Operated Animals into Experimental Groups

Five rats were used in each of the following 5 groups: (1) 3 h following 1 h MCAO, (2) 24 h following 1 h MCAO, (3) rats with a permanently occluded middle cerebral artery 24 h following MCAO, (4) 72 h following 1 h MCAO, and (5) 1 mo following 1 h MCAO. The number of perfused animals included an additional 4 rats for some of the groups (rats at 24 h, 72 h and 1 mo following 1 h MCAO). A further 11 sham-operated rats were used at different time points (11 rats total, 2 for each time point and 3 additional rats for perfusion at 24 h, 72 h and 1 month following MCAO).

**Figure 3:**
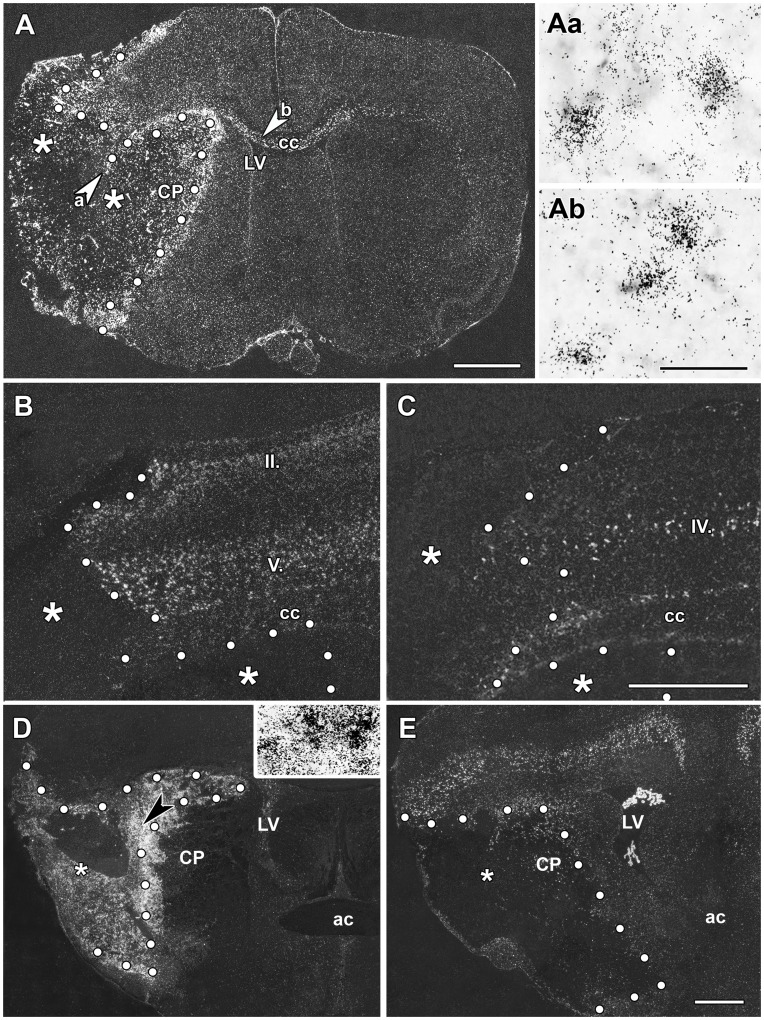
The induction of TGF-β1, - β2, and -β3 mRNA 72 h and 1 mo after MCAO. *In situ* hybridization sections show the expression of TGF-β mRNA. The infarct areas are indicated by the star symbol (*) and the borders of the infarcts are demarcated with white dots. A: TGF-β1 expression is induced at the perimeter of the lesion as well as within the infarct area 72 h after MCAO. High magnification bright field photomicrographs demonstrate that TGF-β1 mRNA accumulates above cell bodies within the infarct area (Aa), as well as in the corpus callosum (Ab). The locations of these pictures are indicated by the arrowheads in the left panel. B: TGF-β2 is present in layers II, III, and V of the cerebral cortex. Only a few cells demonstrate increased expression level of TGF-β2 in the vicinity of the border of the lesion at 72 h after MCAO. C: TGF-β3 is present in some neurons in layer IV of the cerebral cortex, but the level of expression is not higher than that in normal animals D: The induction of TGF-β1 mRNA is demonstrated 1 month after MCAO. TGF-β1 mRNA is abundant within the infarct area except for in a relatively small core region. The field indicated by the large black arrowhead in A is enlarged and shown in bright-field in the inlet to demonstrate that even the densest TGF-β1 signal represents labeling of individual cells as autoradiography grains accumulated above cell bodies. E: TGF-β2 mRNA is present in a few cells at the perimeter of the infarct area. Outside the lesion, TGF-β2 mRNA is distributed in the cerebral cortex as in intact animals but is not induced above normal levels. Abbreviations: ac – anterior commissure, cc – corpus callosum, CP - caudate putamen, LV - lateral ventricle. Scale bar = 2 mm for A and E, 1 mm for C, and 50 µm for B.

**Figure 4:**
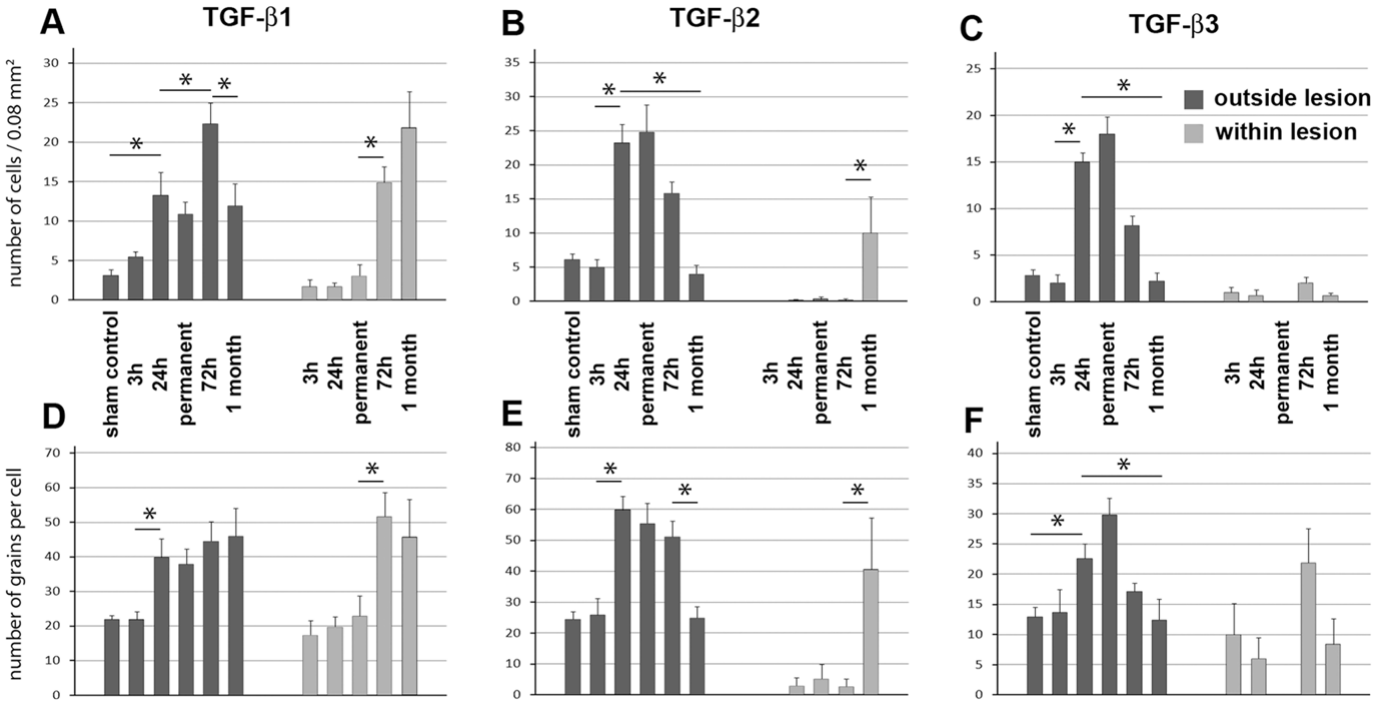
The time course of TGF-β protein expression following MCAO. The numbers of cells within 200 x 400 µm areas (0.08 mm^2^) of coronal brain sections in cortical layer II were counted immediately outside the lesion (dark gray) and 1 mm medial to the border of the lesion within the infarct area (light gray). The number of autoradiography grains was counted above the TGF-β-positive cell nuclei, indicated by autoradiography grain accumulation. Sections from at least 3 brains of the following groups of animals were included in the analysis: sham-operated rats 24 h following MCAO, rats at 3 h and 24 h following 1 h MCAO, rats with a permanently occluded middle cerebral artery 24 h following MCAO, and rats at 72 h and 1 mo following 1 h MCAO. A–C: The number of cells expressing TGF-β1, -β2, and -β3 in 0.08 mm^2^. D-F: The number of autoradiography grains proportional to the mRNA levels of TGF-β1, -β2, and -β3 in single cells. Values at different time points were compared using one-way ANOVA followed by Bonferronís multiple comparisons tests for consecutive time points. The star symbol (*) indicates time points between which the number of TGF-β-expressing cells or the mRNA level of the particular subtype of TGF-β in single cells significantly (p<0.05) changed.

### *In situ* Hybridization Histochemistry

Brains of 33 rats (5 in all 5 groups and 2 sham-operated for each 4 time points) were removed and the fresh tissue was quickly frozen on dry ice, and kept at −80°C. Serial coronal sections (12 µm thick) were cut using a cryostat from bregma level 1 mm to -6 mm [40], mounted on positively charged slides (SuperfrostPlus®, Fisher Scientific, Pittsburgh, PA), dried, and stored at −80°C until use. For *in situ* hybridization, DNA probes developed for the description of TGF-βs in the normal brain were used, which represent 231–647 bp of GenBank accession number NM_021578.2) for TGF-β1, 870–1155 bp of GenBank accession number NM_031131.1 for TGF-β2, and 1046–1343 bp of GenBank accession number NM_013174.1 for TGF-β3 [7]. The primers were chosen to generate probes that recognize all known RNA species for the particular gene. The specificities of the probes were shown by resulting in identical expression pattern as non-overlapping additional probes for TGF-βs [7]. [^35^S]UTP-labeled riboprobes were generated from the DNA probes containing T7 and T3 RNA polymerase recognition sites using a MAXIscript transcription kit (Ambion, Austin, TX). Antisense riboprobes were prepared using T7 RNA polymerase while sense control probes were prepared using T3 RNA polymerase. The preparation of tissue was performed using mRNAlocator Kit (Ambion), according to the manufacturer’s instructions. For hybridization, we used 80 µl hybridization buffer (mRNAlocator Kit; Ambion) and labeled probes of 1 million DPM activity per slide. Washing procedures included a 30 min incubation in RNase A followed by decreasing concentrations of sodium-citrate buffer (pH = 7.4) at room temperature and then at 65°C. Following subsequent dehydration and drying, the slides were dipped in NTB nuclear track emulsion (Eastman Kodak) and stored at 4°C for 3 weeks. Then, the slides were developed and fixed with Kodak Dektol developer and Kodak fixer, respectively, counterstained with Giemsa, and coverslipped with Cytoseal 60 (Stephens Scientific, Riverdale, NJ, USA).

**Figure 5:**
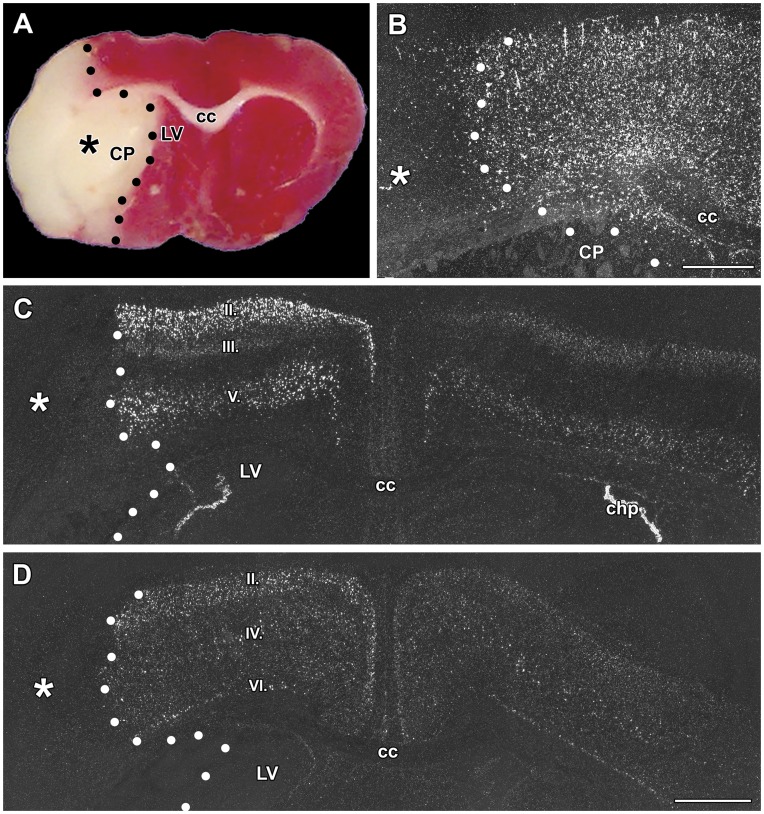
The induction of TGF-β mRNA by 24 h permanent occlusion of the middle cerebral artery. The infarct area is indicated by star symbol (*) and its border is demarcated with black or white dots. **A**: TTC staining demonstrates the effect of ischemia on brain tissue. **B**: TGF-β1 expression is induced in the peri-infarct area of the lesion. **C**: TGF-β2 is induced in layers II, III, and V of the ipsilateral cerebral cortex. The induction is particularly salient in layers II and III where the labeling is much more intense ipsilateral to the lesion compared with the contralateral side. **D**: TGF-β3 is induced in layer II of the ipsilateral cerebral cortex. Abbreviations: cc – corpus callosum, CP - caudate putamen, LV - lateral ventricle. Scale bars = 1 mm for B and D.

### Quantitation of *in situ* Hybridization Data

A cell was considered to express TGF-β if the number of autoradiography grains accumulated in a seemingly Gaussian distribution around a center was at least 3 times higher than the background level in an area corresponding to an average cell size (a circle with a diameter of 25 µm). The background typically contained of 3–4 grains, but always less than 7 grains, per cell in the *in situ* hybridization histochemistry. The number of labeled cells was counted in rectangles (400 µm wide by 200 µm high) along the outside borders of the lesions. Cell counts were confined to specific cortical layers, as well as 1 mm lateral and 1 mm medial to the border of the lesion in layer II of the cerebral cortex (within the lesion and in the ipsilateral healthy tissue, respectively), in the caudate putamen, in the corresponding locations in the contralateral side and in sham-operated animals. In addition, the number of autoradiography grains was counted in 3 randomly selected TGF-β mRNA-expressing cells in each of the 5 rectangles analyzed at different time points after MCAO. Statistical analyses were performed using Prism 5 for Windows (GraphPad Software Inc.). In the first type of analysis, both the number of cells and the number of autoradiography grains measured in different brain regions 24 h following MCAO were compared with the corresponding data obtained from the contralateral hemisphere using Students t-tests. In the second type of analysis, the number of TGF-β mRNA-expressing cells and the number of autoradiography grains measured in these cells in the 6 groups (sham-operated rats 24 h following MCAO, rats at 3 h and 24 h following 1 h MCAO, rats with a permanently occluded middle cerebral artery at 24 h following MCAO, rats at 72 h and 1 mo following 1 h MCAO) were compared using one-way ANOVA followed by Bonferronís multiple comparison post-hoc tests.

**Figure 6:**
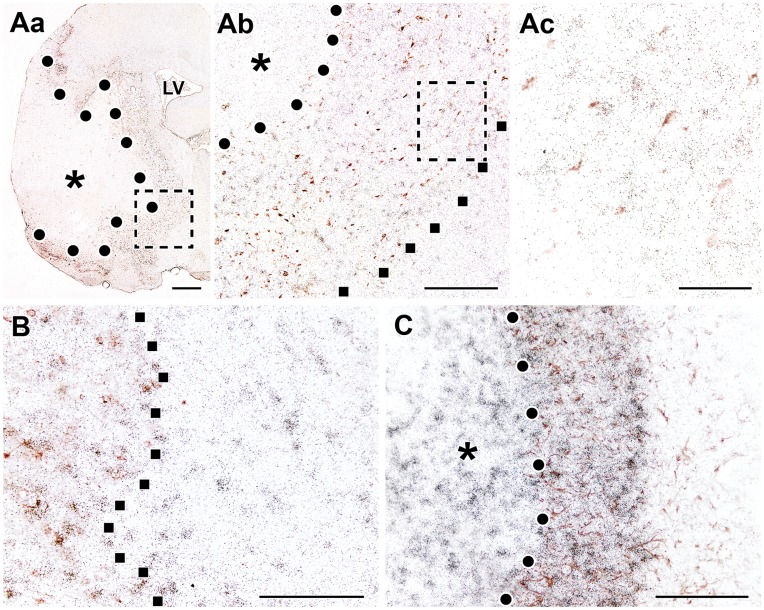
The localization of TGF-β1 and -β2 mRNA in relation to markers of the penumbra and glial scar. **A**: Double labeling of TGF-β1 mRNA (black *in situ* hybridization signal) and immunoreactivity of heat shock protein 70 (Hsp70), a marker of the penumbra (brown precipitate) 24 h following MCAO. The lesion is indicated by star symbols (*), and the lesion border is demarcated by black dots. Black squares demarcate the outer border of the penumbra. The framed area in Aa is shown in Ab to better appreciate the similar distribution of TGF-β1 mRNA and Hsp70 in the penumbra. Ac is a high magnification image of the framed area in Ab and demonstrates that TGF-β1 mRNA and Hsp70 immunoreactivity are not co-localized in the same cells. **B**: Double labeling of TGF-β2 mRNA (black *in situ* hybridization signal) and Hsp70 immunoreactivity (brown precipitate) 24 h following MCAO. Black squares demarcate the outer border of the penumbra. Within the penumbra, the majority of the TGF-β2 mRNA-expressing cells contain Hsp70 immunoreactivity. However, TGF-β2 mRNA expressing cells but not Hsp70-immunoreactive cells are present in the intact brain tissue. **C**: A glial scar can be identified based on the high density of intensely labeled GFAP-positive astrocytes. TGF-β1-expressing cells are present within the glial scar and also within the lesion but not in the intact tissue outside the glial scar. Abbreviations: LV - lateral ventricle. Scale bars = 1 mm for Aa, 400 µm for Ab, 100 µm for Ac, and 200 µm for both B and C.

**Table 1:** Immunolabeling of TGF-β-expressing cells.

	TGF-β1			TGF-β2		TGF-β3
	layer II	layer V	CP	layer II	layer V	layer II
**Number of TGF-β cells/0.08 mm^2^**	18.3±4.9	16.7±3.5	20.5±4.5	18.3±4.2	17.0±3.9	14.0±4.5
**Immunolabeled cells in the % of** **TGF-β cells**						
**NeuN**	3.7±2.8	4.7±3.3	5.7±3.9	87,1±3,8	84,8±2,7	81,1±3.5
**GFAP**	26.3±2.8	21.6±6.2	22.2±4.4	4.2±2.3	4.0±2.1	9.3±5.8
**Iba1**	93.7±3.4	94.0±4.6	96.0±4.1	3.1±2.6	3.5±2.4	6.7±3.4
**Hsp70**	7.2±2.2	4.8±3.9	5.5±2.9	76.3±7.4	69.2±8.9	–
**Fos**	4.1±2.2	4.7±3.3	5.2±2.7	90.2±3.8	91.9±4.6	–
**ATF-3**	8.4±1.9	7.1±2.6	8.5±4.1	2.8±1.9	4.3±2.9	–

Data on TGF-β1 is presented in 3 different locations, layer II and V of the cerebral cortex and the caudate putamen (CP). Because TGF-β2 is not expressed in the caudate putamen, TGF-β2-expressing cells were only counted in the cerebral cortex. Similarly, data on TGF-β3 are provided only for layer II of the cerebral cortex. In the upper row, the total number of TGF-β-expressing cells counted in a 400×200 µm rectangular-shaped area immediately outside the lesion is shown. The number of double-labeled cells was also counted in the same field, and the calculated ratios were averaged. One-way ANOVA did not indicate significant differences between brain regions for any of the markers.

### Cresyl-violet Staining

Parallel series of sections from the MCAO rats were Nissl stained for identification of the border of the lesion. The sections were stained with 0.1% cresyl-violet dissolved in PB, and then differentiated in 96% ethanol containing acetic acid. The sections were dehydrated and coverslipped with Cytoseal 60 (Stephens Scientific).

### Tissue Collection for Immunolabeling

The rats (n = 15; 4 animals following MCAO and a sham-operated control at 3 time points: 24 h, 72 h, and 1 month following the operation) were deeply anesthetized and transcardially perfused with 150 ml of saline followed by 300 ml of 4% paraformaldehyde prepared in phosphate buffer (PB; pH = 7.4). The brains were removed and postfixed in 4% paraformaldehyde for 24 h and then transferred to PB containing 20% sucrose for 2 days. Serial coronal brain sections were cut at 20 µm on a cryostat between 4.0 and −6.0 mm bregma levels. The brain sections were collected in such a way that consecutive sections were mounted on 12 parallel slides. These parallel slides were used for different combinations of *in situ* hybridization and immunohistochemistry to perform the large number of double labeling experiments described below.

**Figure 7:**
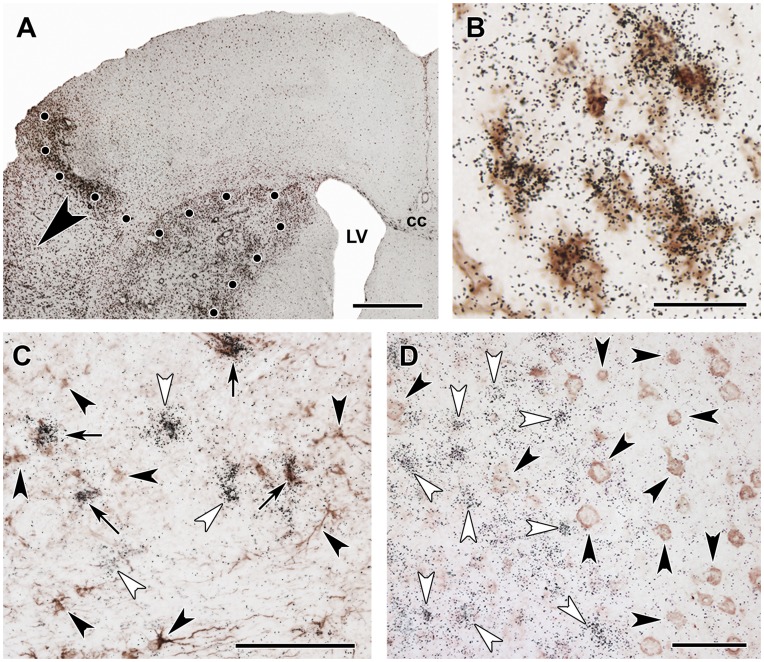
TGF-β1 is induced in glial cells but not in neurons 72 h after lesion. **A**: Double labeling of TGF-β1 mRNA and Iba1 immunoreactivity. The border of the lesion is demarcated by black dots. The position of the high magnification image in B is shown by the large black arrowhead. **B**: A high magnification image shows that the black *in situ* hybridization signal of TGF-β1 is located above Iba1-immunoreactive cell bodies visualized by brown precipitate. **C**: Single labeled TGF-β1 mRNA expressing cells are indicated by white arrowheads, and GFAP-immunoreactive astrocytes by black arrowheads, while double labeled cells are indicated by black arrows. **D**: TGF-β1 mRNA expressing neurons are indicated by white arrowheads, and NeuN-immunoreactive neurons by black arrowheads. No double labeled neurons are present. Abbreviations: cc – corpus callosum, LV – lateral ventricle. Scale bars = 1 mm for A, 30 µm for B, and 100 µm for C and D.

**Figure 8:**
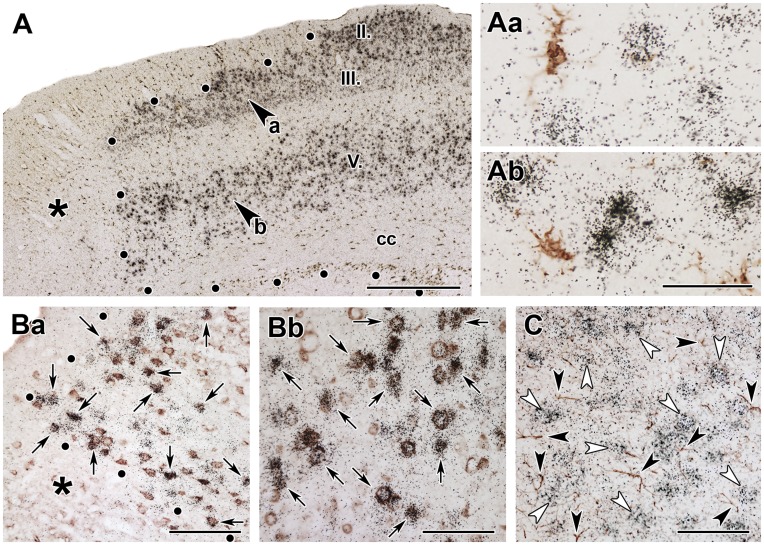
TGF-β2 is induced in neurons but not in glial cells 24 h after MCAO. **A**: Double labeling of TGF-β2 mRNA (black *in situ* hybridization signal) and immunoreactivity of the microglia marker Iba1 (brown precipitate). The lesion is indicated by star symbols (*) and the lesion border is demarcated by black dots. The positions of the high-magnification images in layer III and V of the cerebral cortex are indicated by black arrowheads as Aa, and Ab, respectively. Iba1-immunoreactive microglia do not contain TGF-β2 mRNA in either layer of the cerebral cortex. **B**: Double labeling of TGF-β2 mRNA (black *in situ* hybridization signal) and NeuN-immunoreactive neurons (brown precipitate). Almost all TGF-β2 mRNA-expressing cells contained NeuN immunoreactivity in layer III (Ba), and in layer V (Bb). Examples of double-labeled cells are indicated by black arrows. **C**: TGF-β2 is not expressed in astrocytes. TGF-β2 mRNA expressing neurons are indicated by white arrowheads and GFAP-immunoreactive neurons are indicated by black arrowheads. There are no double-labeled cells present. Abbreviations: cc – corpus callosum. Scale bars = 1 mm for A, 50 µm for Ab, 200 µm for Ba, 100 µm for Bb, and 100 µm for C.

### Immunohistochemistry

Slide-attached sections were pretreated with 3% hydrogen peroxide for 15 min followed by 1% bovine serum albumin in PB containing 0.5% Triton X-100 for 30 min at room temperature. Then, parallel series of sections (every 13th section per brain) were placed in one of the following primary antisera for 24 h at room temperature: rabbit anti-Hsp70 (1∶1000; Abcam, Cambridge, UK, cat. number: ab79852) as a marker of cells in the penumbra, mouse anti-NeuN as a marker of neuronal nuclei (1∶500; Millipore, Billerica, MA, cat. number: MAB377), mouse anti-glial fibrillary acidic protein (GFAP) as a marker of astrocytes (1∶300; Santa Cruz Biotechnology, Delaware, CA, USA; cat. number: sc-33673), rabbit anti-ionized calcium-binding adapter molecule 1 (Iba1) as a marker of microglial cells (1∶1000; Wako, cat. number: 019–197419), rabbit anti-Fos to label activated neurons (1∶25000; Santa Cruz Biotechnology, cat. number: *c-fos* (4) sc-52), and rabbit anti-activating transcription factor-3 (ATF-3) as a marker of neurons with damaged neuronal processes (1∶300; Santa Cruz Biotechnology, cat. number: ATF-3 (C-19): sc-188). Subsequently, the sections were incubated in either biotinylated anti-rabbit secondary antibody (1∶1000; Vector Laboratories, Burlingame, CA, USA) or anti-mouse secondary antibody (1∶1000; Jackson ImmunoResearch, West Grove, PA), for 1 h followed by incubation in avidin-biotin-horseradish peroxidase complex (1∶500; Vector Laboratories) for 1 h. Finally, the sections were treated with 0.06% DAB and 0.003% H_2_O_2_ in Tris hydrochloride buffer (0.05 M, pH = 8.2) for 10 min, and coverslipped with Cytoseal 60 (Stephens Scientific).

**Figure 9:**
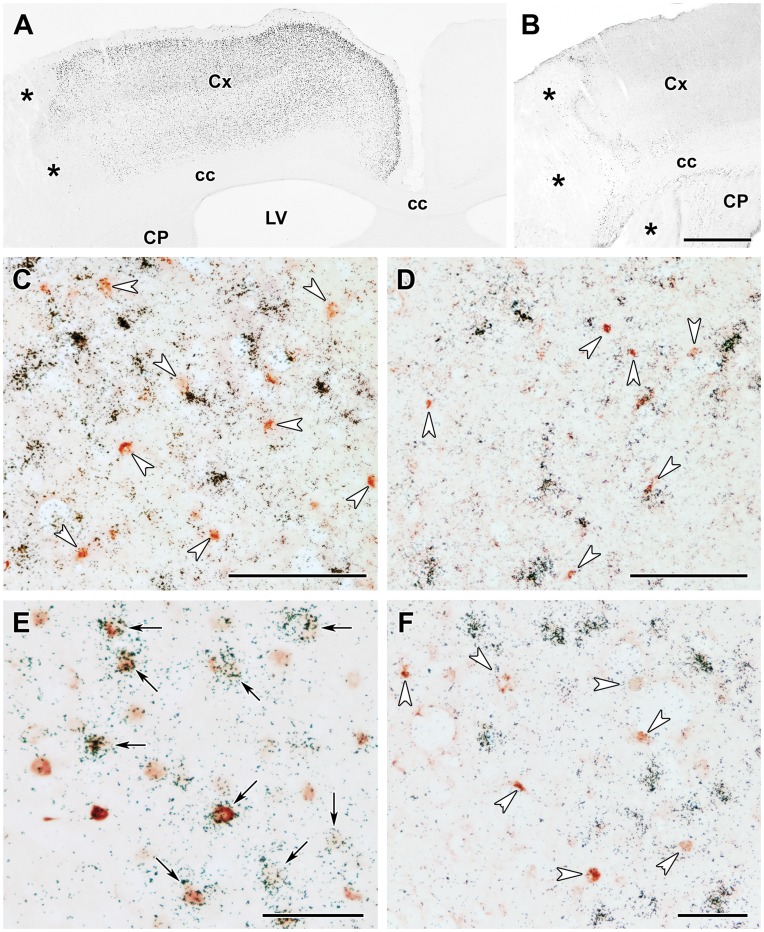
Fos and ATF-3 content of TGF-β expressing cells 24 h after MCAO. **A**: Fos immunoreactive cell nuclei are present in the cerebral cortex ipsilateral but not contralateral to the lesion. The density of Fos-ir cells is particularly high in layers II, III, and V The lesion is indicated by *. **B**: Intensely labeled ATF-3-ir cells are abundant around the lesion. **C**: TGF-β1 mRNA (black grains) does not co-localize with Fos immunoreactivity (brownish-reddish cell nuclei) in the cerebral cortex indicated by white arrowheads. **D**: TGF-β1 mRNA does not co-localize with ATF-3 immunoreactivity (brownish-reddish cell nuclei indicated by white arrowheads). **E**: TGF-β2 mRNA co-localizes with Fos immunoreactivity as black autoradiography grains are located above the brownish-reddish Fos-ir cell nuclei. Double labeled neurons are indicated by black arrows. Some Fos-ir nuclei that do not express TGF-β2 can also be observed. **F**: TGF-β2 mRNA does not co-localize with ATF-3 immunoreactivity (brown cell nuclei). Abbreviations: cc - corpus callosum, CP -caudate putamen, Cx - cerebral cortex, LV - lateral ventricle. Scale bars = 1 mm for B, 100 µm for C and D, and 50 µm for E and F.

The specificity of the antibodies used in the present study has been validated previously. In addition, our own results also argue for specific labeling. The anti-Hsp70 antiserum (Abcam, Cambridge, UK, cat. number: ab79852) resulted in an intense western blot band at 70kDa according to the manufacturer. In addition, we did not observe any Hsp70 labeling in the intact brain. In turn, Hsp70 immunoreactivity appeared in the peri-lesion area following MCAO as expected as it is a marker of the penumbra [36], [37]. The anti-NeuN antibody (Millipore, Billerica, MA, cat. number: MAB377) labeled neurons and was confirmed by their morphology. This antibody has been used in several previous publications that required the validation of its specificity [41]. The anti-GFAP antibody (Santa Cruz Biotechnology, Delaware, CA, USA; cat. number: sc-33673) labeled a single band with the appropriate molecular weight of approximately 50 kDA in western blot experiments according to the manufacturer. In our study, the anti-GFAP antibody labeled cells with morphology characteristic of astrocytes. In addition, GFAP immunoreactivity markedly increased in cells surrounding the lesion at the time points when astroglial scar formation was expected. The anti-Iba1 antiserum (Wako, cat. number: 019–197419) labeled cells with morphology characteristic of resting microglia with ramified thin processes, as well as large, ameboid-shaped activated microglia in and around the lesions. In addition, this antiserum has been extensively used as a marker of microglial cells in previous studies that required the validation of its specificity [42]. The anti-Fos antiserum (Santa Cruz Biotechnology, cat. number: *c-fos* (4) sc-52), a marker for labeling activated neurons, did not label cells in the sham-operated rats, except for in a few restricted brain regions, in our study. However, as expected, labeled neurons appeared in the cerebral cortex following ischemic attack. In addition, this antiserum has been used before as a marker of activated cells in previous studies that required the validation of its specificity [43]. The anti-ATF-3 antiserum (Santa Cruz Biotechnology, cat. number: ATF-3 (C-19): sc-188) did not label cells in the sham-operated rats, except for in a few brain regions, in our study. However, as expected, labeled neurons appeared around the lesion, as ATF-3 is a marker used for labeling neurons with damaged neuronal processes. This antiserum has been used before as a marker of damaged cells in previous studies that required the validation of its specificity [44].

### Combination of Immunohistochemistry and *in situ* Hybridization Histochemistry

Slide attached sections of perfused brains were first processed for *in situ* hybridization, as described above. Thus, tightly bound RNA-RNA pairs were already formed by the time immunohistochemistry was performed, immediately before dipping the slides into autoradiographic emulsion. In addition, the solutions used for perfusion and immunohistochemistry were prepared with DAPC-treated RNAse-free water, which ensured that the labeling intensity of the *in situ* hybridization histochemistry did not decrease significantly. The immunolabeling protocol was the same as that used for single labeling immunohistochemistry. Immunoreactivity was visualized using DAB reactions, after which the *in situ* hybridization procedure was continued by dipping the slides into emulsion. Each double labeling experiment included controls, which went through the double labeling procedure without application of radioactive *in situ* hybridization probes. These controls demonstrated that the DAB signal did not induce an autoradiography signal.

### Analysis of Double Labeling Experiments

A cell was considered TGF-β-expressing if it met the criteria described above for single label *in situ* hybridization histochemistry. A TGF-β-expressing cell was considered immunopositive if at least half of the area of the circle containing the accumulation of autoradiography grains contained immunoreactivity for a particular marker. For comparison, the number of TGF-β-expressing cells and the number of double-labeled cells (TGF-β + NeuN, GFAP, Iba1, Hsp70, Fos, or ATF-3) were counted in 400×200 µm rectangular areas at the outside borders of the lesions in layers II and V of the cerebral cortex and in the caudate putamen. The percentage ratios of double-labeled TGF-β-expressing cells were compared between different brain regions using one-way ANOVA.

### Histological Analysis and Image Processing

The sections were examined using an Olympus BX60 light microscope in both dark-field and bright-field. Images were captured at 2048×2048 pixel resolution with a SPOT Xplorer digital CCD camera (Diagnostic Instruments, Sterling Heights, MI) using a 4×objective for dark-field images, and 4–40×objectives for bright-field images. The contrast and sharpness of the images were adjusted using the “levels” and “sharpness” commands in Adobe Photoshop CS 8.0. Full resolution was maintained until the photomicrographs were printed, at which point the images were adjusted to a resolution of 300 dpi.

## Results

All 3 types of TGF-βs were induced in the rat brain following MCAO. We describe the specific topographical mRNA expression of each type of TGF-β in response to focal ischemia at different time points following MCAO and the different cell types that express TGF-βs. We demonstrate that TGF-β2 appears in neurons that also show Fos activation following MCAO in the ipsilateral cerebral cortex but not in neurons expressing ATF-3.

### The Expression Patterns of TGF-β mRNAs in the Brain Following MCAO

**3** **h after transient MCAO:** A 1 h occlusion of the middle cerebral artery resulted in a lesion that was histologically visible 3 h after the onset of the occlusion. The lesioned tissue lost its structure in the lateral part of the caudate putamen as observed using Giemsa counterstaining (Fig. 1A). TGF-βs were not present within the infarct area but TGF-β1 was induced in the area around the lesion. The induction of TGF-β1 was striking in the non-lesioned area of the caudate putamen (Fig. 1B), as TGF-β1 is not expressed in this area in sham-operated animals (not shown). TGF-β1 also appeared in the cerebral cortex. The most intense labeling was present lateral to the lesion in the insular and frontal somatosensory cortices. In contrast, the distribution of TGF-β2 and -β3 mRNA did not change compared with intact and sham-operated animals at this early time point. Furthermore, induction of these 2 subtypes was not observed (Fig. 1C).

**24** **h after transient MCAO:** A lesion that includes a large part of the caudate putamen as well as a large part of the ipsilateral cerebral cortex was found 24 h after MCAO. TGF-β1 mRNA was detected in the peri-infarct area around the lesion in all cerebral layers (Fig. 1D) as well as in the caudate putamen (Fig. 1E). A significant increase in the level of TGF-β1 mRNA, compared with the corresponding contralateral brain regions of layers II-VI of the cerebral cortex and the caudate putamen, was observed (Figs 2A,D). TGF-β2 was also induced around the infarct area in response to MCAO in the cerebral cortex but not in the caudate putamen (Fig. 1F). A quantitative analysis of gene expression revealed that in layer II, both the number of TGF-β2-expressing cells and the level of TGF-β2 mRNA increased. The number of TGF-β2-expressing cells, but not the TGF-β2 mRNA levels in individual cells, increased in layer III In contrast, in layers V and VI, which have significant basal TGF-β2 expression, the mRNA levels increased in cells that already expressed TGF-β2, without the recruitment of additional cells (Figs. 2 B, E). IIVII Induction of TGF-β3 was visible only in a few cells in layer II of the cerebral cortex in the vicinity of the lesion (Figs. 1G, 2C,F). The expression levels of any of the TGF-βs were not higher contralateral to the lesion than in sham-operated or intact animals.

**72** **h after transient MCAO:** At 72 h after MCAO, TGF-β1 expression remained elevated around the lesion (Fig. 3A) with a further significant increase in the number of TGF-β1-expressing neurons when compared with 24 h after MCAO (Fig. 4A). In addition, a number of TGF-β1 mRNA expressing cells appeared within the infarct area and in the corpus callosum (Fig. 3A). In parallel, the levels of TGF-β2 and -β3 mRNA showed a tendency to return to the intact levels of expression in the cerebral cortex (Fig. 3B,C). In contrast to TGF-β1, TGF-β2 and -β3 mRNA did not appear within the ischemic lesion (Figs. 3B,C, 4).

**1 month after transient MCAO:** The area of the infarct had shrunk 1 month after MCAO. TGF-β1 expression remained very high within the infarct area but the number of TGF-β1-expressing cells was reduced outside of the infarct (Figs. 3D, 4A). TGF-β2 and -β3 were no longer induced in the cerebral cortex but their pre-lesion level of expression was retained (Fig. 3E). Some TGF-β2-expressing cells appeared within the lesioned area (Fig. 4B,E) and at the perimeter of the lesion.

**Permanent MCAO (24** **h):** Permanent occlusion of the middle cerebral artery resulted in large lesions that included the majority of the ipsilateral caudate putamen and cerebral cortex (Fig. 5A). Concurrently, the induction of TGF-βs became more pronounced than following transient MCAO. TGF-β1 mRNA occupied a larger area around the lesion than was observed 24 h after 1 h MCAO (Fig. 5B). TGF-β2 induction remained restricted to layers II, III, and V of the cerebral cortex. TGF-β2 expression was induced in these layers in the vicinity of the lesioned area and was enhanced up to the midline in the ipsilateral cerebral cortex (Fig. 5C). Nevertheless, an induction of TGF-β2 mRNA was not found in the contralateral hemisphere. A similar induction pattern of TGF-β3 was observed in layer II of the ipsilateral cerebral cortex. In contrast, the cells that express TGF-β3 in layers IV and VI in the intact rats did not display any increase in TGF-β3 mRNA levels (Fig. 5D).

### Distribution of TGF-βs in Relation to the Penumbra and Astroglial Scar

Heat shock protein 70 immunoreactivity, indicative of the penumbra, was distributed in cell bodies around the lesion 24 h following transient MCAO but was absent within the lesion and the intact brain tissue (Fig 6A). TGF-β1-expressing cells had a similar distribution at this time point (Fig. 6A). However, TGF-β1 mRNA and Hsp70 immunoreactivity were typically not present in the same cells (Table 1).

The distribution of TGF-β2 and Hsp70 overlapped within the penumbra 24 h following MCAO. However, additional TGF-β2-expressing cells were present in the intact brain tissue in layers II, III, and V of the cerebral cortex (Fig. 6B). Interestingly, approximately 75% of the cells expressing TGF-β2 within the penumbra also contained Hsp70 (Table 1).

A glial scar was present around the lesion 1 month following MCAO. The scar tissue was visualized by intense GFAP-immunolabeling (Fig. 6C). TGF-β1-expressing cells were abundant within the glial scar and the infarct area but were virtually absent in the intact tissue (Fig. 6C). The majority of the TGF-β1-expressing cells within the glial scar did not contain GFAP (Fig. 6C). TGF-β2 and –β3 were not present in astrocytes forming the scar tissue 1 month following MCAO (not shown).

### The Cell Types Expressing TGF-βs in Response to Transient MCAO

In the brain sections of intact and sham-operated rats, Iba1 immunohistochemistry labeled resting microglia with ramified thin processes. At the perimeter of the lesion, intensely labeled Iba1-positive, large, ameboid-shaped cells were present 24 h after MCAO suggesting the appearance of activated microglia. At 72 h after MCAO, Iba1 immunoreactivity was further increased around the lesion. In addition, Iba1-immunoreactive (Iba1-ir) cells were visible within the infarct area (Fig. 7A). The distribution of Iba1-ir cells was similar to that of TGF-β1-expressing cells at each time point. Furthermore, a combination of Iba1 immunohistochemistry and TGF-β1 *in situ* hybridization indicated co-localization of Iba1 and TGF-β1 within the ischemic core as well as around the lesion (Fig. 7B). Approximately 95% of the TGF-β1 cells contained Iba1 immunoreactivity (Table 1). In contrast, the distribution of TGF-β2- and -β3-expressing cells had different distributions (Fig. 8A) and showed only minimal co-localization with Iba1 (Table 1).

GFAP- and NeuN-ir cells were not present in the ischemic core, but indicated the extent of the lesion by their presence in the intact tissue. Approximately 25% of the TGF-β1-expressing cells around the lesion were also labeled by GFAP (Fig. 7C) while only a very small percentage of TGF-β2 (Fig. 8C) and-β3-expressing cells contained GFAP immunoreactivity (Table 1). Over 80% of TGF-β2 and –β3 mRNA-expressing cells co-localized with the NeuN-positive cells suggesting that these subtypes are expressed in neurons (Fig. 8B). In turn, TGF-β2 and –β3 were not present in microglial cells as concluded from their absence in Iba1-ir cells (Fig. 8A).

### The Induction of Fos and ATF-3 in Neurons Expressing TGF-βs after Transient MCAO

Fos-expressing neurons were present throughout the cerebral cortex ipsilateral to the lesion 24 h after MCAO. However, the distribution of Fos-ir cells showed some topographical organization. The highest density of Fos-ir cells was observed in layer II while layers I, IV, and VI contained only a few Fos-ir cells (Fig. 9A). In contrast, intensely labeled ATF-3-ir cells were present only around the lesion (Fig. 9B). We did not observe Fos or ATF-3 immunoreactivity in TGF-β1 mRNA expressing cells (Fig. 9C,D). In contrast, almost all of the examined TGF-β2-expressing neurons contained Fos immunoreactivity (Table 1), while other Fos-ir cells did not express TGF-β2 (Fig. 9E). The area containing ATF-3-ir cells only slightly overlapped with the area containing TGF-β2-expressing cells. In these regions, ATF-3 immunoreactivity did not substantially co-localize with TGF-β2 (Fig. 9F).

## Discussion

Our findings indicate that TGF-βs are induced in the brain following focal ischemic attack and that the mRNAs of the different types of TGF-βs have individual topographical distributions in distinct cell types. We compare these patterns to the distributions of previously published expressional data following MCAO, and also provide additional information on the mechanisms involved in the induction of these factors. Finally, our data are discussed in terms of the potential functions of TGF-βs in brain ischemia.

### The Expression of the Different Subtypes of TGF-βs in the Brain Following Focal Ischemia

The *in situ* hybridization probes were designed to recognize all known forms of mRNA for each type of TGF-β. Therefore, the *in situ* hybridization signals we obtained represent the sum of the different alternatively spliced forms of each TGF-β that were present in the tissue [45]. The results confirmed and expanded previous findings related to the induction of TGF-β1 in response to MCAO. A biphasic expression of TGF-β1 mRNA has been reported following MCAO in rats [29]. In this study [29], *in situ* hybridization of TGF-β1 was analyzed using film autoradiograms, which does not allow the spatial resolution of our study. Nevertheless, an early induction of TGF-β1 in the peri-infarct cerebral cortex was followed by the induction of TGF-β1 within the infarct. We confirmed these observations and also revealed an early phase induction of TGF-β1 in the peri-infarct caudate putamen. Furthermore, we demonstrated that the peri-infarct localization of TGF-β1 is restricted to the penumbra. However, it must be emphasized that our data demonstrate only the induction of TGF-β mRNA. In the future, a validation of these results at the protein level will be important to fully appreciate the potential role of TGF-βs in ischemia.

The expression of TGF-β1 in microglia following MCAO has previously been suggested using a combination of immunohistochemistry for OX42 and *in situ* hybridization for TGF-β1 [33]. We used Iba1, an established marker of both ramified and activated microglia [46], to demonstrate the expression of TGF-β1 in microglia. In contrast to the absence of TGF-β1 in astrocytes reported by Lehrmann et al (1998), we observed TGF-β1 in some GFAP-positive neurons confirming studies on the induction of TGF-β1 in astrocytes following transient ischemia [47]. In addition, we used the neuronal marker NeuN to demonstrate that TGF-β1 is not expressed in neurons, suggesting that the induction of TGF-β1 is restricted to glial cells. The small percentage of apparent co-localization could be the result of single labeled cells situated above each other in different planes. Furthermore, we first described the expression patterns of TGF-β2 and -β3 following focal ischemia and compared them to that of TGF-β1. A marked difference was found; TGF-β2 and -β3 were exclusively induced in specific layers of the cerebral cortex. In those layers, however, the induction took place even in regions remote from the lesion and outside of the penumbra in the ipsilateral cortex. The level of TGF-β2 increased in cells that already had basal TGF-β2 expression in layer V but also in newly recruited cells in layer II and III of the cerebral cortex. The induction patterns of TGF-β2 and -β3 were not biphasic; rather they peaked at 24 h following MCAO and subsequently decreased. Another major difference between the induction of TGF-β1 and -β2 is that the latter was exclusively expressed in neurons. Thus, the present study largely expanded our knowledge regarding the induction of TGF-ßs following MCAO, as it is the first to describe the expression of all 3 types of TGF-βs at different time points following an ischemic attack.

### Possible Mechanisms of Induction of TGF-ßs

As the induction of all 3 types of TGF-ßs became more pronounced by permanent occlusion, as opposed to 1 h occlusion followed by reperfusion, it is likely that ischemia itself rather than reperfusion evokes the induction of TGF-ßs. The precise overlap between the area of TGF-ß1 expression and the border of the penumbra also suggests that ischemia itself leads to the induction of TGF-ß1. Focal ischemic damage is known to activate microglia in the infarct area, as well as in the adjacent surviving area [32] via inflammatory cytokines released from ischemic neurons and invading blood cells [48], [49]. The expression of Iba1, which is probably involved in the mobility and phagocytosis of microglia [50], is enhanced after transient focal ischemia in microglia and in macrophages that infiltrate the infarct area [51]. However, microglial activation predominates over macrophage infiltration following MCAO [52]. Therefore, it is likely that the increased expression of TGF-ß1 is predominantly a consequence of microglial activation, which is also supported by the appearance of TGF-ß1 in the peri-infarct area. In fact, inflammatory cytokines such as tumor necrosis factor-alpha and IL-1 have been shown to induce TGF-ß1 expression in microglia and astrocytes [53], [54].

The neuronal induction and the markedly different expression patterns of TGF-β2 and -β3 from that of TGF-β1 suggest different mechanisms of induction for these subtypes. The mechanisms of induction were addressed using double labeling of TGF-β2 mRNA with ATF-3 and Fos immediate early genes. Damage to axons was shown to induce the activation of ATF-3 in the cell nuclei [55]. MCAO resulted in a very fast induction of ATF-3 within the infarct area followed by the appearance of ATF-3 in the peri-infarct area [56] that co-localized with neurons but not glial cells [57]. However, the lack of co-localization of ATF-3 with TGF-βs suggests that axonal damage is not as major factor in the induction of TGF-βs. In contrast, TGF-β2-expressing cells had a distribution similar to that of the *c-fos* activation pattern following MCAO [58], [59] suggesting a previous activation of these neurons [60], [61]. Furthermore, the TGF-β2-expressing cells all contained Fos immunoreactivity. As Fos expression can be caused by cortical spreading depolarization following MCAO [62], it is likely that the induction of TGF-β2 and -β3 occurs by cortical spreading depolarization, possibly *via c-fos* activation. In cortical layers II and V, some neurons in intact tissue expressed TGF-β2. It is plausible that cortical spreading depolarization enhances the expression levels of TGF-β2 in these neurons. However, the recruitment of additional neurons is likely as TGF-β2 and TGF-β3-expressing neurons also appear in layers III and II, respectively.

### Functional Implications of the Induction of TGF-βs Following Focal Ischemia

TGF-βs have neuroprotective actions against hypoxic events as demonstrated in different experimental model systems [16]–[19]. The different spatial and temporal patterns of TGF-β induction following MCAO support their participation in distinct functions after focal ischemic attack. TGF-β1 expression was elevated in the ischemic penumbra, a major target of neuroprotective treatment [63]. TGF-β1 may exert negative auto-feedback inhibitory action on the function of microglia, which are required to not only remove debris but also contribute to neuronal damage by releasing oxygen species and inflammatory cytokines [64]. TGF-β1 can deactivate microglia [65] and selectively promote their apoptosis [66]. TGF-βs may also participate in other processes following ischemia including neoangiogenesis [67], [68] and glial scar formation [14]. Local injection of a TGF-β antagonist into cerebral wounds reduced glial scarring [35] and abolished fibrinogen-induced effects on glial scar formation [69], possibly by affecting the proliferation, migration and activation of astrocytes [8], [70] and the extracellular matrix environment [71].

A direct neuroprotective function of the TGF-βs is also likely based on their *in vitro* effects on neuronal survival [9], [13], [18]. These actions may be mediated by different neurotrophic factors whose actions are modulated by TGF-βs [10], [72], [73]. After ischemic lesions, the restoration of neural functions may also require novel neurite growth and synapse formation, processes that are also influenced by the TGF-βs [74]–[76]. Many of these actions were examined by techniques that did not differentiate between the TGF-β subtypes. However, our results suggest separate functions for the TGF-β subtypes. TGF-β2, co-expressed with Fos in the cerebral cortex may be involved in the regulation of neuronal apoptosis as cortical neurons are known to exhibit programmed cell death following MCAO [77]–[80] and Fos expression is thought to be a crucial part of the programmed cell death pathway and whose inhibition was shown to be neuroprotective after MCAO [81]. Given that TGF-β2 and -β3 inhibit hypoxia-induced neuronal apoptosis [9], [82], it is possible that induced cortical TGF-β2 and -β3 exert neuroprotective functions by inhibiting apoptosis in the ipsilateral cerebral cortex. In addition to neuronal survival, TGF-βs might also be involved in neuronal repair, a process increasingly investigated for post-stroke treatment [83]. Ischemia induced neural stem cell proliferation and differentiation involves TGF-β pathways [84]. In turn, TGF-βs increased the expression of neuronal markers in cortical primary cell cultures [27], as well as during adult neurogenesis [26].

### Conclusions

Endogenous TGF-β1, -β2 and -β3 are expressed in brain tissue following a focal ischemic lesion caused by MCAO. However, significant differences exist between the spatial and temporal patterns of expression of TGF-β subtypes. Furthermore, the TGF-β subtypes are expressed in separate cell types, and co-localize with different immediate early genes, suggesting that their inductions following an ischemic attack requires distinct mechanisms. These results imply that the different subtypes of TGF-βs participate in different aspects of neural tissue protection.
